# Prevalence of challenge-proven IgE-mediated food allergy in infants in the Barwon Region, Victoria, Australia

**DOI:** 10.1186/2045-7022-5-S3-P89

**Published:** 2015-03-30

**Authors:** John Molloy, Jennifer Koplin, Anne-Louise Ponsonby, Mimi L K Tang, Fiona Collier, Katrina Allen, Peter Vuillermin

**Affiliations:** 1 Child Health Research Unit, Barwon Health, Geelong Hospital, Victoria, Australia; 2 Murdoch Children's Research Institute, Parkville, Australia; 3 School of Medicine, Deakin University, Waurn Ponds, Australia; 4 University of Melbourne, Parkville, Victoria, Australia; 5 Department of Allergy and Immunology, Royal Children's Hospital, Parkville, Victoria, Australia

## Background

A recent study found that approximately 10% of 1 year old infants living in Melbourne, Australia have challenge proven food allergy; which is the highest reported rate in the world. The prevalence of IgE-mediated food allergy among Australian infants living outside a major metropolitan city has not been measured.

## Objective

To determine the prevalence of IgE-mediated food allergy in a population-derived cohort of infants aged 1 year from the Barwon Region, in the south east of Australia.

## Methods

The Barwon region, located approximately 100 km south of Melbourne, is centred around the city of Geelong and has metropolitan, rural and coastal communities. The Barwon Infant Study (BIS) is a population-derived birth cohort study (n = 1069 infants). As part of the 12 month review infants undergo skin prick testing to five foods: cow's milk, raw egg, peanut, cashew and sesame. Those with any detectable wheal size are offered a formal in-hospital oral food challenge using a validated protocol to determine their clinical allergy status.

## Results

To date 671/717 (93%) of infants who have completed their one-year BIS review have undergone skin prick testing. Overall 88/671 (13.1%; 95% CI, 10.6-15.9) were sensitized to one or more of the five foods: raw egg 9.5% (95 % CI, 7.4-12.0), peanut 3.2% (95% CI, 2.0-4.9), cashew 2.2% (95% CI, 1.2-3.6), cow's milk 1.1% (95% CI, 0.0-2.0) and sesame 0.4% (95% CI, 0.004-1.3).

67 of 88 (76%) infants with allergic sensitization have undergone a food challenge. Among those challenged, the overall rate of proven food allergy is 43/67 (64%; 95% CI, 51.5-75.5). For each specific food the rates are: raw egg 37 (68.5%; 95% CI, 54.4-80.4), peanut 6 (46.1%; 95% CI, 19.2-74.8), cashew 1 (16.6%; 95% CI, 0.4-64.1), sesame 1 (50%; 95% CI, 12.5-98.7) and cow's milk 2 (50%; 95%CI, 6.7-93.2).

Based on these preliminary figures the estimated prevalence of challenge proven food allergy among the BIS cohort is 6.4% (95% CI, 4.6-8.5). The estimated allergy rates for specific foods are: raw egg 5.5% (95 % CI, 3.9-7.5); peanut 0.8% (95% CI, 0.3-1.9); cashew 0.1% (95% CI, 0.00-0.8); sesame 0.1% (95%CI, 0.00-0.8) and cow's milk 0.2% (95% CI, 0.0-1.0).

## Conclusions

Preliminary results suggest that the prevalence of food allergy in a regional centre in Australia may be less than in a major metropolitan centre such as Melbourne. Further investigation is underway to verify this regional difference and to identify contributing factors.

